# Knowledge Gaps in Gluten-Free Diet Awareness among Patients and Healthcare Professionals: A Call for Enhanced Nutritional Education

**DOI:** 10.3390/nu16152512

**Published:** 2024-08-01

**Authors:** Paula Crespo-Escobar, Maialen Vázquez-Polo, Maria van der Hofstadt, Concepción Nuñez, Miguel A. Montoro-Huguet, Itziar Churruca, Edurne Simón

**Affiliations:** 1 i+HeALTH Strategic Research Group, Department of Health Sciences, Miguel de Cervantes European University (UEMC), 47012 Valladolid, Spain; crespoescobar.paula@gmail.com; 2 Department of Nutrition and Obesity, Hospital Recoletas Campo Grande, 47007 Valladolid, Spain; 3 GLUTEN3S Research Group, Department of Nutrition and Food Science, University of the Basque Country, UPV/EHU, 01006 Vitoria-Gasteiz, Spain; maialen.vazquez@ehu.eus; 4 ALINUA, Food and Nutrition Cabinet Health Science Faculty, University of Alicante, UA, 03690 Alicante, Spain; mariavdhr@gmail.com; 5 Laboratorio de Investigación en Genética de Enfermedades Complejas, Hospital Clínico San Carlos, Instituto de Investigación Sanitaria del Hospital Clínico San Carlos (IdISSC), 28040 Madrid, Spain; conchita.npardo@gmail.com; 6 Gastroenterology, Hepatology and Nutrition Unit, University Hospital San Jorge, 22004 Huesca, Spain; maimontoro@gmail.com; 7 Department of Medicine, Faculty of Health and Sport Sciences, University of Zaragoza, 22002 Huesca, Spain; 8 Aragon Health Research Institute (IIS Aragon), 50009 Zaragoza, Spain

**Keywords:** knowledge GFD, GFD follow-up, adherence to gluten-free diet, CeD patients, CeD healthcare professionals, dietitian–nutritionist’s role, patient association

## Abstract

Diet is the only treatment for celiac disease (CeD), and good adherence to a gluten-free diet (GFD) is the only way to ensure complete remission and to prevent complications. Limited education about the disease and a GFD is an attributing factor to inadequate adherence. Thus, our aim was to assess the current knowledge about a GFD and the clinical monitoring of adherence to the diet among CeD people and HCPs. Specific questionnaires were designed and distributed to assess the knowledge of CeD people (Q1 questionnaire) (*n* = 2437) and to analyze the follow-up of the disease from the perspective of patients (Q2 questionnaire) (*n* = 1294) and HCPs (Q3 questionnaire) (*n* = 346). Two-thirds of HCPs specialized in pediatric care, while one-third did so in adult care. In CeD people, general questions regarding food classification and cross-contamination are well understood. When patients have doubts, 51.4% reported using the Internet and social networks. Thus, it is crucial that resources like social media are reliable and provide valuable information. Q3 revealed the lack of time to follow up the diet after diagnosis (48% of HCPs allocate < 15 min), the interest in further training, and the need for a professional specialized in diets within the healthcare system. In conclusion, it is essential to enhance nutritional education to increase awareness of a GFD.

## 1. Introduction

Celiac disease (CeD) is a chronic immune-mediated disorder that affects approximately 1% of the general population. CeD is characterized by inflammation of the small intestinal mucosa and subsequent villous atrophy, triggered by the ingestion of gluten protein. Gluten ingestion leads to several intestinal (e.g., diarrhea, abdominal pain) and extraintestinal (e.g., osteoporosis) symptoms in patients with CeD. If left untreated, CeD can lead to serious complications, including intestinal cancer or infertility [1,2]. The only available treatment is a strict, lifelong, gluten-free diet (GFD), which should result in complete symptomatic, histological, and serological remission, and prevent these complications [3]. However, it can be exceedingly difficult to completely avoid all gluten-containing foods. Thus, adherence to a GFD among people with CeD is estimated to range from 42% to 91% in adults [4], and from 23% to 98% in children and adolescents [5], depending on the population considered and the criteria used to define adherence. The key to the success lies in dietary counseling by a specialized dietitian–nutritionist and in the maintenance of adherence to the prescribed diet by the patient [6].

Several studies have examined the factors associated with adherence to a GFD and the most often reported are cognitive (knowledge, attitudes, understanding of product labels, and other food intolerances); emotional (anger, depression, anxiety); and sociocultural and sociodemographic characteristics (public awareness, eating out, travel, social events, and cost of gluten-free foods); as well as joining an advocacy group and having access to a regular dietary follow-up [4,7,8]. Limited education about the disease and a GFD among CeD patients is also an attributing factor to inadequate adherence [7,9,10]. Additionally, the management of healthcare professionals (HCPs) might influence the adherence of patients to this diet. Many CeD patients express dissatisfaction with the time dedicated and quality of information provided by their physicians regarding a GFD, leading them to seek information on social networks [11]. Therefore, it has been widely demonstrated that achieving good adherence to a GFD requires two main issues [12]: (1) that HCPs dedicate sufficient time to explain the diet after diagnosis, that they stay constantly updated on the diet, and that they have practical tools to measure adherence during the follow-up. This control allows them to detect and correct any errors and transgressions in the diet and (2) that patients and their families have comprehensive counseling and nutritional education about a GFD. They must be informed about changes in their food habits and lifestyle, and be taught about how to integrate a GFD into all spheres of their life [13,14].

There are some guidelines that outline the essential information that patients should receive to correctly follow a GFD. These include explaining the disease and the requirement for a lifelong GFD, planning a balanced GFD, discussing the benefits of adhering to a GFD and the risk of nutritional deficiencies, identifying sources of hidden gluten in various food items and critical points of cross-contamination, educating patients on how to read labels before purchasing the gluten-free food, providing precautions while eating out and traveling, and ensuring access to celiac support groups and resources [15,16].

Thus, the aim of our study was to assess the current knowledge about a GFD and the clinical monitoring of adherence to the diet among CeD patients and HCPs in Spain in order to design improvement strategies in the training of patients and professionals..

## 2. Materials and Methods

### 2.1. Study Design and Instruments

Specific questionnaires were designed to assess the knowledge of the celiac population, and their caregivers, regarding CeD and a GFD (Q1, questionnaire 1). Additionally, the follow-up of the pathology in clinical settings was analyzed from the perspectives of patients or their relatives (Q2, questionnaire 2) and HCPs (Q3, questionnaire 3). The questionnaires were developed with inputs from gastroenterologists, registered dietitian–nutritionists, and representatives of patients’ associations. Surveys were created for online filling out and included multiple answer choices to ensure the maximum accuracy in the responses.

Q1 and Q2 were intended for individuals with CeD or people who are responsible for the care of those with CeD (such as parents or guardians). They were distributed online among CeD patient association members of FACE (Spanish Federation of Celiac Societies). On the one hand, the Q1 survey contained 3 general questions on sources of information about CeD and a GFD and 14 questions to measure the knowledge of a GFD among people with CeD, mainly in relation to the gluten content of different food types and cross-contact. On the other hand, Q2 was designed by researchers from the Spanish Society of Celiac Disease (SEEC). It comprised 20 questions and was divided into 5 subsections: sociodemographic questions (4 items), information obtained from HCPs about a GFD (3 items), inquiries about sources of information (3 items), details about the follow-up to ensure dietary compliance (5 items), and questions related to knowledge about a GFD (5 items).

The Q3 questionnaire was also designed by the SEEC to be answered by HCPs working with CeD patients, both pediatric and adult. It was distributed online among scientific societies related to CeD, gastroenterology, and nutrition in Spain. The Q3 questionnaire for HCPs was composed of 22 questions, divided into 3 subsections: sociodemographic background (3 items), clinical practice related with diagnosis and follow-up and questions regarding the explanation of a GFD during the follow-up (11 items), and inquiries related to knowledge about a GFD (8 items).

All the questionnaires were distributed throughout 17 Spanish autonomous communities and sampling was carried out by the snowball method. In order to ensure greater dissemination, they were also shared through the different social network platforms (Facebook, Instagram, X) of FACE and their member associations. Additionally, HCPs distributed the questionnaires among their CeD patients, aiming to reach non-member patients as well. Before the start of the study, all participants agreed to take part in it. The study was submitted to the Ethics Committee for Human Research of the University of the Basque Country, UPV/EHU (M10_2023_303). This committee established that this research does not require evaluation by the Ethics Committee for human subjects, given the anonymized data fall outside the scope of the General Data Protection Regulation (GDPR).

### 2.2. Statistical Analysis

The study of frequencies and percentages was used to conduct the descriptive analysis. Chi-square tests were used to compare the qualitative responses between groups. Results were considered as statistically significant with a *p*-value less than 0.05 (95% confidence interval). Participants who did not complete the entire questionnaire were excluded from the analysis. IBM SPSS Statistics for Windows, version 28.0. (IBM Corp., Armonk, NY, USA), was used for the statistical analysis of the data.

## 3. Results

### 3.1. Knowledge of Celiac Population Concerning a GFD

**Figure 1:**
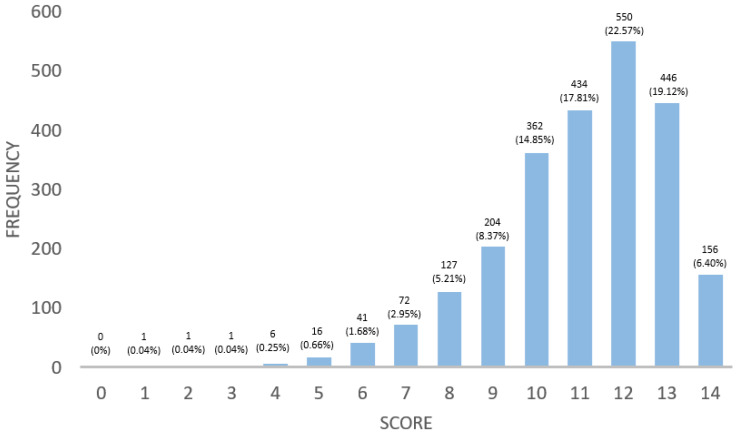
Distribution of scores obtained from questions measuring GFD knowledge among individuals with CeD.

The Q1 questionnaire involved 2437 people with CeD. Out of these participants, 2036 (83.5%) reported that they were members of a patient association. The remaining respondents cited various reasons for not being members: 252 (10.3%) cannot afford it, 94 (3.9%) believe they no longer need it, and 55 (2.3%) consider it to be of no use. Participants were asked, “Who explained to you what you know about CD?” In response, 1267 subjects (52%) said it was the doctor who diagnosed them, 1053 participants (43.2%) credited celiac associations, 62 (2.5%) mentioned a private nutritionist, 51 (2.1%) said the practice nurse, and 4 participants (0.2%) obtained information from other sources. When asked where they turn to for information about a GFD, 1253 participants (51.4%) reported using the Internet and social networks, 759 (31.1%) turned to the patient association, 371 (15.2%) consulted their doctor, 51 (2.1%) sought advice from a dietitian–nutritionist, and 3 (0.1%) looked for information through other means. Participants answered 14 questions to measure their knowledge of a GFD (Table A1). Knowledge was assessed on a scale of 0–14 according to the number of correct answers. The average score was 11.06 ± 1.97 points. The distribution of the scores is illustrated in Figure 1.

The average total score varied based on who provided the information about CeD and a GFD. Statistically significant differences were observed between those who received the information from the doctor who diagnosed them and those who received it from the association (*p* < 0.001). Those who received information through associations achieved higher scores (10.81 ± 2.02 points vs. 11.36 ± 1.83 points, respectively).

### 3.2. Follow-Up of a GFD in Clinical Settings

#### 3.2.1. The Healthcare Professional’s Perspective

To begin, descriptive issues of clinical practice need to be detailed. The Q3 questionnaire was distributed among multidisciplinary HCPs related to CeD. It involved 346 multidisciplinary HCPs: primary care pediatricians (*n* = 125; 36.1%); gastroenterologists (42.8%) either for adult (*n* = 66) or pediatric (*n* = 82) patients; family physicians (*n* = 47; 13.6%); nurses (*n* = 9; 2.6%); dietitians–nutritionists (*n* = 6; 1.7%); and other HCPs (*n* = 11; 3.2%). Two-thirds of HCPs were specialized in pediatric care, while one-third were in adult care.

Of these respondents, 83.2% reported diagnosing between 0 and 25 cases of CeD per year while 11% diagnosed between 25 and 50 cases annually. Regarding the follow-up care, 61.8% provide it to 0–25 people with CeD, while 38.2% indicated monitoring more than 25 patients.

Participants were queried about how much time they typically spend explaining a GFD to patients during the diagnostic visit, and 91% indicated a duration of less than half an hour. Of these, 166 individuals (48%) allocate less than 15 min, while 143 (41.3%) spend between 15 and 30 min. Conversely, 31 professionals (9%) dedicate between 30 and 60 min with only 6 (1.7%) extending beyond 60 min. Despite this, the majority of the respondents (*n* = 276; 79.8%) expressed a desire for more time in consultation to thoroughly guide patients in adhering to a GFD. Regarding the time spent on follow-ups to measure the adherence to a GFD in patients, it was found that 290 individuals (83.8%) reported devoting less than 15 min. Additionally, 48 (13.9%) stated they spent between 15 and 30 min. Only a small fraction, six individuals (2.3%), reported spending between 30 and 60 min.

There were noticeable differences in the time in consultation, whether for diagnosis or follow-up, depending on the age of the patients. In this regard, the time spent explaining a GFD after diagnosis was related to the type of patient treated (*p* < 0.001; Cramer’s V = 0.23), with a higher percentage of professionals dedicated to children in the categories denoting more time (Table 1). Similarly, the time spent explaining a GFD during follow-up also showed a statistically significant association with the type of patient treated (*p* = 0.014; Cramer’s V = 0.16) (Table 1). Curiously, during the follow-up, the percentage of HCPs spending 30–60 min with adults was higher than with children. This could be related to the persistence of symptoms and the ongoing effort to identify their underlying causes.

The willingness of HCPs to spend more time explaining a GFD was related to gender (*p* = 0.005; Cramer’s V = 0.18), with women requesting more time (84.5% in women compared to 69.2% in men), and to the age of the professionals (*p* = 0.047; Cramer’s V = 0.15). Professionals in the younger age groups, specifically those up to 50 years of age, requested the most time.

Regarding the recommendations to visit a dietitian–nutritionist, 145 (41.9%) of respondents do not recommend such visits, while a similarly sized group (*n* = 146; 42.2%) said they sometimes suggested it. Merely 15 (4.3%) indicated recommending it to half of their patients and 40 (11.6%) always give this advice. Interestingly, recommendations vary depending on the age of the patients, with a higher tendency to endorse it for adults than for children (*p* < 0.001; Cramer’s V = 0.25).

Concerning the recommendations to join a patient association, 300 HCPs (86.7%) point out that they always advise it after the diagnosis, 31 (9.0%) say they suggested it sometimes, and 15 (4.3%) never recommend it. Interestingly, the recommendation to join a patient association was only mentioned after the initial diagnosis, but not during follow-up visits.

When asked where they direct their patients when they have doubts about a GFD, the majority of respondents (*n* = 316; 91.3%) recommend consulting the local celiac association. Additionally, 166 (48.0%) suggest visiting specific websites, and 116 (33.2%) refer patients to scientific societies. Only 100 (28.9%) endorse visiting a dietitian–nutritionist, 30 (8.7%) to their reference general practitioner, 19 (5.5%) to others, and 3 (0.8%) do not give any advice.

To continue, the quality of consultation needs to be addressed. Regarding adherence to a GFD, only 41 (11.8%) HCPs claimed to use specific nutritional tools like nutritional surveys to assess adherence. A majority (63.3%) mentioned using general, open-ended, non-specific questions, while a significant number of participants (20.2%) do not ask their patients about adherence-related issues.

**Figure 2:**
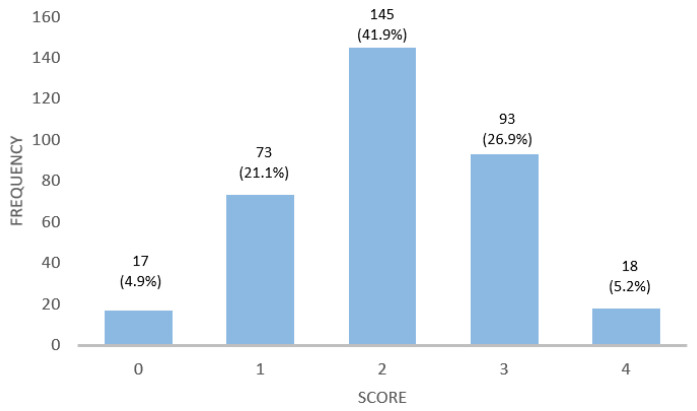
Distribution of scores obtained from questions measuring GFD knowledge among healthcare professionals.

As far as the HCP’s knowledge about a GFD is concerned, participants answered four questions to measure their knowledge of a GFD (Table A3). Knowledge was assessed on a scale of 0–4 according to the number of correct answers. The average score was 2.06 ± 0.94 points. The distribution of the scores is illustrated in Figure 2.

Noticeably, 61 (17.6%) of HCPs believe that quinoa and amaranth may contain gluten and 245 (70.8%) believe that the declaration of gluten-free traces is mandatory. Moreover, approximately 15% do not know more than three critical points where cross-contamination might occur or they cannot specify any at all.

In terms of knowledge and information about a GFD, a meaningful 96% of participants considered it relevant to have access to specific information, training courses, and materials. When they have specific doubts regarding a GFD, 235 (67.9%) look for the information in national and international scientific societies, 182 (52.6%) mention they use specific medical websites, 71 (20.6%) browse their doubts on the Internet and 63 (18.2%) do so in specific divulgation blogs about CeD, 25 (7.2%) use specific resources from the specialized food industry, and 27 (7.8%) use other references. The point here is that only 131 (35.0%) turn to the patient associations and consider them as an interesting partner.

In addition, the vast majority (93.4%) stated that the national health system should incorporate more dietitians–nutritionists to better assess patients with specific dietary needs. Small differences were observed when considering the age of HCPs (*p* = 0.007; Cramer’s V = 0.16). Nearly all HCPs under 50 years of age supported this incorporation (98.1%), compared to 90.0% of those over 50 years.

#### 3.2.2. The Patient’s Perspective

A total of 1294 individuals participated in the Q2 questionnaire, ranging in age from 6 to 80 years (mean = 40.65; SD = 13.15). Of the respondents, 16.9% (*n* = 219) identified as men, 82.3% (*n* = 1065) as women, and 0.8% (*n* = 10) preferred not to disclose their gender. Among the participants, 67.5% (*n* = 873) reported being diagnosed with CeD, while 32.5% (*n* = 421) were first-degree relatives of someone with this disease. The age at first diagnosis ranged from 9 months to 72 years, with an average age of 10.42 years (SD = 17.28).

To begin, descriptive issues of managing a GFD need to be detailed. Regarding the first steps to follow a GFD, the majority of the respondents (*n* = 924; 71.4%) agreed that their first recommendations about the diet were provided by their physician. Additionally, 182 (14.1%) reported receiving guidance from the local celiac association, 34 (2.6%) from a dietitian–nutritionist, and 13 (1%) from their nurse. Furthermore, it should be noted that 141 (10.9%) cited other sources, with friends/partners/family members (*n* = 62) and self-study (*n* = 51) being the most notable.

However, opinions about the quality of the information provided about a GFD for the first time were diverse. Approximately 415 (32.1%) considered it poor, while 204 (15.8%) found it sufficient. On the optimistic side, 354 (27.4%) regarded it as good, and 321 (24.8%) deemed it very good. Apart from that, it is noteworthy that 908 (70.2%) of respondents had never consulted with a dietitian–nutritionist.

When asked about the sources of information they rely on when they have doubts about a GFD, local patient associations emerged as the preferred choice/option for 804 (62.1%) of respondents. Additionally, 306 (23.7%) consulted their family physician, while 106 (8.2%) sought advice from dietitians–nutritionists. Furthermore, 100 (7.7%) expressed confidence in the information provided by scientific societies. In terms of social and familial networks, 230 (17.8%) sought guidance from specific blogs or influencers, while 149 (11.5%) relied on other sources such as family, friends, colleagues, and consultation groups formed on digital media platforms (like Facebook and WhatsApp). The Internet, in general, was the second most utilized source of information for addressing doubts about following a GFD, with 759 (58.7%) of participants referring to it. Differences were detected in the frequency of use of this tool: 264 (20.4%) use it infrequently, 594 (45.9%) occasionally, 96 (7.4%) monthly, and 294 (22.7%) use it weekly.

When it comes to the quality of their GFD, 1082 (83.6%) believed they maintained a healthy diet, while 142 (11.0%) were unsure, and 70 (5.4%) considered their diet to be unhealthy. This positive perception may be correlated with the responses to the question about visiting a dietitian–nutritionist for advice, as only 34.6% of celiac patients answered affirmatively.

Concerning oat consumption, a considerable percentage (68.2%, *n* = 882) abstain from consuming oats altogether. Among those who do consume oats, the majority opt for certified gluten-free varieties. Of the latter, 342 (26.4%) partake of/eat oats occasionally, while 62 (4.8%) include them in their daily diet. Fortunately, only a small minority (0.6%) consume oats without confirming whether they are certified gluten-free.

**Figure 3:**
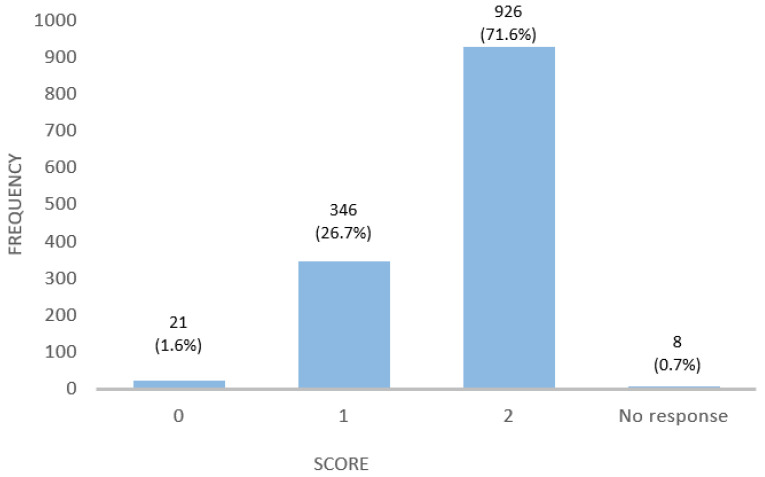
Distribution of scores obtained from questions measuring GFD knowledge among patients.

They were also asked questions about the different food groups to assess the risk of gluten contamination and the subsequent risk of transgression of a GFD. Participants answered two questions, and this knowledge was assessed on a scale of 0–2 according to the number of correct answers. The average score was 1.70 ± 0.49 points. The distribution of the scores is illustrated in Figure 3. A total of 74.5% of participants demonstrate the ability to identify gluten-free food staples based on their natural absence of gluten. A higher percentage, 95.4%, identified foods prone to contamination, although it is true that queried foods were described within the tables provided by celiac associations (Table A2).

To continue, the quality of consultation needs to be addressed. When querying about follow-up medical appointments, our focus was on assessing if these visits inquired about adherence to a GFD and the level of compliance with it. Significant statistical differences were observed depending on whether the responses were provided by the patients themselves or their first-degree relatives (*p* < 0.001; Cramer’s V = 0.19). While 69% of patients with CeD responded positively, this percentage escalated to 86.2% when family members were surveyed. Moreover, it was asked whether these follow-up visits involve a thorough nutritional assessment for the patient, including evaluations of weight, height, and body composition, as well as specialized complete blood tests aimed at evaluating vitamin and mineral levels. Again, significant statistical differences were observed depending on the group being asked (*p* < 0.001; Cramer’s V = 0.36). The majority of patients with CeD responded negatively (*n* = 524; 60%), indicating that they did not undergo this assessment, while 102 (24.2%) family members similarly reported that such an evaluation was not conducted. Similarly, patients stated unequivocally (95.3%) that they did not have their food intake recorded to assess the nutritional quality of their diet. Finally, the patient’s caregivers interviewed also expressed a more positive evaluation regarding the perceived knowledge of HCPs conducting follow-up on a GFD (*p* < 0.001; Cramer’s V = 0.26). Table 2 illustrates the obtained answers.

#### 3.2.3. Differences and Similarities between the Perspectives of CeD Patients and HCPs

The perception of the need to visit a dietitian–nutritionist varied between patients and HCPs. Among the patients, 386 (29.8%) believed it was necessary to see a dietitian–nutritionist, whereas 201 HCPs (58.1%) considered such visits essential. This indicates that a significantly higher percentage of professionals recognized the requirement of consulting a diet specialist (*p* < 0.001; Cramer’s V = 0.24). In contrast, both groups agreed on the need for more specific training of HCPs on a GFD. A total of 1242 patients (96.0%) and 332 professionals (96.0%) considered this training necessary.

## 4. Discussion

The primary objective of this study was to evaluate the knowledge about the GFD of people with CeD, as well as of HCPs involved in diagnosing and treating this condition. Additionally, this study evaluated the clinical approaches used for diet adherence, assessing the perceptions of both patients and HCPs. Based on the results obtained, two blocks can be discussed as follows: understanding of a GFD and compliance to the diet. Both aspects have a direct impact on adherence to a GFD.

Currently, EU Regulation 1169/2011, which came into force in 2014, permits foods that naturally do not contain gluten to be labeled as “gluten-free.” However, no official regulation specifies which foods are considered naturally gluten-free. To address this, the Association of European Coeliac Societies (AOECS) has developed a classification system defining three categories: generic foods (naturally gluten-free), conventional foods (naturally gluten-free but potentially contaminated during processing), and specific foods (produced without gluten under conditions ensuring maximum safety). This classification, endorsed by all patient associations in Europe, is crucial as it facilitates the easy and safe categorization of foods. Consequently, understanding this classification can be regarded as enhanced understanding for patients and their families [17].

In this context, the results according to the survey conducted by FACE (Q1) indicate that the general questions on food classification and cross-contamination are well understood, with over 78% of celiac respondents answering these questions correctly. However, when specific questions about the safety of certain foods are asked, there is a higher rate of incorrect answers. For these specific food-related questions, the correct response rate is only 62%. Regarding questions about medications in the survey designed for patients and/or their relatives, 20% expressed doubts, and more than 15% incorrectly believe that CeD patients are not a risk group for vaccination. It is noteworthy that individuals who are members of a patient association tend to have higher rates of correct answers. This is important because previous research has shown that patients who belong to celiac associations or groups have more knowledge and greater adherence to a GFD, as they receive more emotional and social support [14,18,19]. Therefore, the role of associations in ensuring proper adherence to a GFD is crucial for patients and has been widely recognized in earlier studies [9,20]. These results are consistent with those obtained from the Q2 survey targeting CeD patients or their relatives, where three out of every four respondents knew how to identify gluten-free products well, and the overwhelming majority were able to identify cross-contamination risks. This indicates a high degree of patient knowledge in these two critical areas of a GFD.

In contrast, a study by Paganizza et al. in Italy rated CeD patients’ knowledge about the gluten content of foods as poor, with only 1 out of 104 participants (0.96%) answering all questions correctly [14]. Compared to that, in our study, 156 of 2437 participants (6.4%) answered correctly to all questions in Q1. The study conducted in Italy emphasized the association of the knowledge of CeD people about a GFD with the adherence to the diet, suggesting the promotion of educational and behavioral programs [14]. Comparable results were obtained by Sahin et al. in Turkey, where they observed that none of the CeD participants answered all questions correctly, in a knowledge questionnaire, highlighting significant gaps in knowledge [21]. Similarly, Riznik et al. found that patients scored an average of 56.4% correct on a CeD knowledge questionnaire, indicating a widespread lack of understanding [13]. Additionally, Pohoreski et al. found that 63% of adolescents with CeD were not sufficiently trained about a GFD [22]. Furthermore, a recent systematic review carried out by Abu-Janb analyzed the facilitators and barriers to adherence to a GFD among adults with CeD at various levels: individual, interpersonal, organizational, community, and systemic. This research demonstrated that at the individual level, knowledge of the disease and/or a GFD was the most significant factor identified in the literature. Specifically, fourteen studies reported that the lack of knowledge was a barrier to GFD adherence while up to eight studies identified a good level of awareness is a facilitator [7]. The authors emphasized the importance of patients receiving correct nutritional education about a GFD to prevent this lack of knowledge from being a barrier to gluten-free adherence. These findings agree with those of our study.

Another cross-sectional study conducted by Muhammad et al. analyzed the association between receiving a GFD prescription and understanding food labeling with adherence to a GFD [23]. They revealed that a misunderstanding of food labels was significantly associated with a poorer gluten-free dietary adherence CDAT score. More precisely, 73% of those who reported not comprehending food labels were classified as not adhering to a GFD, compared to 45% who understood food labels. Although we did not specifically analyze adherence to a GFD, we did assess knowledge related to food labeling. Based on Muhammad’s findings, we anticipate that patients who make errors in labeling questions may exhibit poorer adherence to the diet. Improving knowledge in this area could potentially enhance adherence [23].

In relation to a GFD and its follow-up, between 70% and 52% of patients, in both the Q1 and Q2 surveys, indicate that information about a GFD is given by their physician after diagnosis, with almost half considering that the information received was scarce or just sufficient. These facts are relevant because, in the survey aimed at HCPs (Q3), there are questions with a high percentage of errors on the basic aspects of a GFD. For instance, almost one out of five of the HCP respondents mistakenly believed that pseudocereals like quinoa and/or amaranth may have gluten, and only 13.3% were aware that the declaration of gluten traces is not mandatory. These data highlight the need for improved knowledge about a GFD. This necessity is further emphasized by the limited time spent explaining the diet, with more than 90% of HCPs dedicating less than half an hour to this task after the diagnosis. Knowledge about the diet and the time dedicated to it are two fundamental areas that professionals should focus on to enhance patient adherence to treatment. Moreover, it is important to emphasize that almost all (96%) of the HCPs demand more training, indicating their perception of needing to increase their knowledge of a GFD. These results are consistent with others reported in different studies that have shown that one of the major pitfalls is their dissatisfaction with the extent and quality of information provided by their physicians [11,12,20]. In addition, Ukkola et al. [11] reported that patients were more satisfied with the counseling provided by a dietitian–nutritionist than that provided by physicians. The information provided after the CeD diagnosis was deemed inadequate in 28% of cases by physicians and 12% of cases by dietitians. The primary reasons for patient dissatisfaction were scant information (59% for physicians and 20% for dietitians) and insufficient counselor training (7% and 18%, respectively). These data align with our findings, where 50% of patients consider that they received poor information, reinforcing the idea of including dietitians–nutritionists in the ongoing care of celiac patients.

Riznik and coworkers also analyzed HCPs’ knowledge about CeD in Central Europe [13]. The authors concluded that this level of understanding is unsatisfactory given that, on average, only half of the questions were answered correctly. Although this study focuses more on knowledge about the disease in general and the diagnosis rather than a GFD specifically, the findings are comparable and can be extrapolated to our study, where comprehension about a relevant aspect of a GFD is low among professionals [13]. Other published studies support these outcomes and underline the importance of enhancing nutritional programs among HCPs [24,25,26]. In contrast, despite our study revealing a lack of knowledge among HCPs and their own demand for more training, the perception of patients during follow-up appears more positive than at the time of diagnosis. Specifically, 43% of celiac people and 40% of caregivers consider the level of knowledge of their physician to be acceptable, while 44% of caregivers rate it as very good. This perception is influenced by the fact that caregivers of minors with CeD, who are typically followed by pediatricians, responded to the survey. Pediatricians, as noted in our survey, demonstrate better accuracy in GFD-related questions. In this regard, Sahin et al. also found that pediatric gastroenterologists were the physicians who responded best to the questionnaire, with a score of approximately 66 out of 100 [21]. Similar findings were obtained by Riznik et al., who observed that pediatric gastroenterologists obtained the highest scores on the knowledge questionnaire and it was speculated that this may be associated with a greater awareness about the burden of CeD [13]. It is also plausible that over the course of follow-up, patients may acquire more knowledge about a GFD. Consequently, they might perceive HCPs as more knowledgeable, since they have fewer questions that need answering compared to the time of diagnosis.

While it is encouraging to note this positive result, it is important to acknowledge the findings from studies such as Ukkola et al., which emphasize the critical nature of the information provided about a GFD at the time of CeD diagnosis compared to that during follow-up. Ukkola’s study showed that physicians’ attitudes and the guidance given at diagnosis significantly influenced patients’ experiences with the disease and their adherence to treatment after one year. Poor doctor–patient communication and scant information at diagnosis were associated with shock reaction, disapproval, and a negative attitude towards both the disease and the diet [11].

Finally, regarding the search for knowledge, another important aspect is where patients seek information about a GFD. In the Q1 survey, more than half of the respondents (51.4%) reported looking for information on the Internet and social networks when they have questions about the diet, while 30% consult their local association. This can be explained by the immediacy the Internet provides for resolving doubts. Other studies, such as the one conducted in Italy in 2016, indicated that 37% of participants used the Internet for information, with this percentage increasing to 45% among those who demonstrated adequate adherence to a GFD [14]. A more recent study conducted in 2020 showed increased use of this resource, indicating that 96% of celiac patients and their families in the Saudi Arabian Celiac Patient Support Group (SCPSG) used social networking platforms to manage their disease [27]. The use of this resource was notably high, with 76.4% of respondents consulting it daily [27]. These figures are significantly higher compared to the usual use described in Q2, where only 22.7% consult it weekly and nearly half use it only occasionally. In the SCPSG survey, the majority of respondents acknowledged that social media was helpful in increasing their understanding of the disease and their adherence to a GFD. More precisely, 78% of participants considered social media effective in raising community awareness of celiac disease, a finding similar to that found by us in a previous cross-sectional study published recently [28]. Tomlin et al. concluded that the Internet significantly influences parental knowledge of CeD. However, they emphasized that accurate information from specialists is essential to alleviate anxiety related to the use of a GFD [29]. This information is relevant because it opens a new source of information about a GFD that will have to be managed from the professionals’ consultation and include, as part of the dietary advice, where to look for reliable information on the Internet. However, for this to happen, it is essential that HCPs are also aware of these resources and able to validate information from the Internet and social media sources as well.

Continuing with the resources to improve knowledge and resolve doubts, while three-fifths of patients (62.1%) turn to the celiac association to resolve their doubts, only one-third of HCPs utilize this resource, even though they mostly recommend going to an association after diagnosis. This disparity is noteworthy because patient associations are becoming increasingly professionalized and staffed by dietitian–nutritionists and psychologists, as well as professionals with specialized postgraduate training. These resources, currently underutilized by HCPs, can serve as valuable allies or stakeholders for HCPs in addressing the disease.

The survey results concluded that most of the HCPs stated that the National Health System should incorporate more dietitians–nutritionists for stronger dietary monitoring and compliance with the specific dietary needs of patients. In this sense, a recent review highlighted the usefulness of the clinical follow-up of the diet by a specialized dietitian–nutritionist since, among other advantages, the early detection of transgressions actually results in cost savings for the healthcare system [16]. Interestingly, this perspective contrasts with the fact that HCPs often do not recommend their patients visit a dietitian or nutritionist. It is plausible to suggest that this inconsistency is due to the belief that such services should be covered by the healthcare system rather than being the financial responsibility of the patient. This fact is corroborated by a work carried out in Spain that evaluates the integration of dietitians–nutritionists into multidisciplinary teams across primary, specialized, and public healthcare, which reveals a low or virtually non-existent implementation at the state level [30].

Educational programs can help to improve the detected gaps by first identifying the real concerns, requirements, uncertainties, and challenges faced by CeD individuals and HCPs [28]. Next, the type of educational program should be tailored to the target audience described. Similarly, it is essential that those delivering nutrition education have adequate training, highlighting the role of dietitians–nutritionists. Regarding the methodology, it has been proven that group-based educational programs are successful in improving both gastrointestinal symptoms and overall quality of life [31]. In the case of children, parental involvement in the program is essential [32]. Nutritional education can be delivered through face-to-face sessions or online, as significant results have been published through virtual formats [33,34,35]. Finally, e-learning is effective in improving the comprehension of a GFD in children and their families [36], and it has also been proposed as a useful tool for HCPs [13,21].

The strength of this study lies in assessing knowledge about gluten-free foods and diet for monitoring CeD. It aims to assess a GFD in terms of both knowledge and clinical practice. In addition, the high number of participants, which reached 3731 among CeD patients and their relatives, adds robustness to the results. Moreover, the substantial participation of HCPs from various specialties, covering both adult and pediatric patients, further strengthens this study. A noteworthy aspect of this study is the parallel consideration of both patient and HCP perspectives in diet follow-up, enabling a comparison between them. However, there are some weaknesses. The attempt to limit the number of questions led to incomplete coverage of both perspectives in certain areas. These surveys were conducted nationwide in Spain; therefore, the conclusions may not be applicable to other healthcare systems or cultural contexts. Another limitation of this study may be the potential self-reporting bias in the questionnaire responses, particularly with regard to the knowledge and practices of healthcare professionals. Data collection through FACE and its associations may introduce bias, as many respondents are linked to patient associations. Previous studies have shown that members of these associations are more familiar with and adhere more closely to the dietary guidelines. Additionally, when information is provided by a family member, it is often assumed the patient is a pediatric case, although this cannot be confirmed categorically because this information was not specifically requested.

## 5. Conclusions

The knowledge of the celiac population and their caregivers regarding gluten-free foods is insufficient to ensure correct adherence to a GFD and achieve the nutritional balance of the diet. From the perspective of HCPs, the very limited time available during consultations, along with the need for additional specialized training, may explain the lack of knowledge among healthcare providers and their restricted ability to monitor adherence to a GFD. HCPs agree that this task should be carried out by dietitians–nutritionists, but referrals to these diet specialists are recommended only on a limited basis probably due to their minimal presence in public healthcare. Patient associations frequently fill this gap, but patients and caregivers often resort to less reliable sources of information, such as the Internet and social networks, when they have doubts. A fundamental option is to enhance nutritional education, not only for patients but also for clinicians, and to reinforce the social networks consulted to ensure that the information disseminated is reliable and scientifically based.

## Figures and Tables

**Table 1:** Time spent in consultation by HCPs, in diagnosis, and follow-up, depending on the age of the patients usually treated.

		Type of Patients		
		Children *n* = 213	Adults *n* = 133	Total HCPs *n* = 346
How much time do you spend explaining the GFD after reporting the diagnosis?	<15 min *	39.9%	60.9%	48.0%
	15–30 min *	49.3%	28.6%	41.3%
	30–60 min	9.9%	7.5%	9.0%
	>60 min	0.9%	3.0%	1.7%
How much time do you spend explaining the GFD at follow-up?	<15 min	85.0%	82.0%	83.8%
	15–30 min	14.6%	12.8%	13.9%
	30–60 min *	0.5%	5.3%	2.3%

Significant differences between types of patients are identified with * (*p* < 0.001).

**Table 2:** Perception of the HCPs’ knowledge about the GFD of CeD patients and their relatives.

Knowledge Level of HCPs		CeD Patient (*n* = 873)	First-Degree Relative/Caregiver (*n* = 421)
None	% of total	4.9%	0.5%
Very little	% of total	29.3%	14.3%
Acceptable	% of total	43.2%	40.6%
Very good	% of total	22.6%	44.7%

## Data Availability

The original contributions presented in the study are included in the article; further inquiries can be directed to the corresponding author/s.

## Appendix A

**Table A1:** Results of the questionnaire Q1 to measure the knowledge about the gluten-free diet (GFD) of people with celiac disease distributed by FACE (*n* = 2437).

Question		Number of Responses	Percentage (%)
What is celiac disease?			
	An autoinmune disease	1906	78.2
	An intolerance	460	18.9
	An allergy	41	1.7
	None of the above	30	1.2
Do you know what a generic product is?			
	A product that is naturally gluten-free	1967	80.7
	A product that may contain gluten	363	14.9
	A product that contains gluten	66	2.7
	A product specially formulated for people with CD	41	1.7
Do you know what a conventional product is?			
	A product that is naturally gluten-free	175	7.2
	A product that may contain gluten	1904	78.1
	A product that contains gluten	314	12.9
	A product specially formulated for people with CD	44	1.8
Do you know what a specific product is?			
	A product that is naturally gluten-free	55	2.3
	A product that may contain gluten	105	4.3
	A product that contains gluten	57	2.3
	A product specially formulated for people with CD	2220	91.1
What should we look for in order to know if a product is gluten-free?			
	In the gluten-free claim	18	0.7
	It is included in the list of FACE foodstuffs	196	8.0
	It has a registered Crossed Grain Trademark	164	6.7
	All are correct	2059	84.5
Do you know the difference between the gluten-free claim and the registered Crossed Grain Trademark?			
	The gluten-free claim complies with the labelling law and the Crossed Grain Trademark not only complies with the law but also with the ELS certification standards	297	12.2
	The gluten-free label is not safe and the Crossed Grain Trademark is	46	1.9
	The gluten-free claim is managed solely by the company and the Crossed Grain Trademark requires an external audit	74	3.0
	A and C are correct	2020	82.9
What is the food list?			
	A publication produced by FACE with information provided by manufacturers and compiling gluten-free products	2393	98.2
	A business that FACE does with companies	9	0.4
	An instruction book	8	0.3
	I do not know	27	1.1
Do you know what cross-contamination is?			
	When a gluten-containing product comes into contact with a gluten-free product.	2430	99.7
	Something that only happens in bars	3	0.1
	Something that never happens	1	0.0
	I do not know	3	0.1
In what situations would there be cross-contamination?			
	Spreading butter on toast	3	0.1
	Sharing a deep fryer in which all kinds of products are made	189	7.8
	When passing bread between people at the table	7	0.3
	All are correct	2238	91.8
What type of product is the pre-cut fruit?			
	A conventional	897	36.8
	A specific	173	7.1
	A generic	1298	53.3
	A banned	69	2.8
What kind of spices are conventional?			
	Grain and leaf	774	31.8
	Ground	1007	41.3
	None	117	4.8
	All	539	22.1
What happens with lentils?			
	That care must be taken with the ingredients that are incorporated into the stew and we must ensure that they are all gluten-free	180	7.4
	That they are contaminated in the field and should be consumed dry by sieving before cooking	540	22.2
	That they are naturally gluten-free	190	7.8
	All of the above are correct	1527	62.7
Should people with celiac disease be careful with medicines?			
	Can medicines contain gluten?	36	1.5
	Information on excipients must be found on the packaging of medicines in order to use those that do not contain gluten	298	12.2
	Information on the gluten content of medicinal products can be obtained from sections 2 and 6 of the package leaflet and from pharmacists	136	5.6
	B and C are correct	1963	80.5
	No response	4	0.2
Do we have to be vaccinated against the flu?			
	Vaccines are useless	9	0.4
	There is no need because we are not sick	193	7.9
	Yes, we are considered a risk group	2016	82.7
	Only people over 65 and pregnant women	200	8.2
	No response	19	0.8

## Appendix B

**Table A2:** Results of the questionnaire Q2 to measure the knowledge about the GFD of patients distributed by SEEC (*n* = 1294).

Question		Number of Responses	Percentage (%)
Olive oil, unseasoned packaged olives, maize, quinoa and natural yoghurt are all foods that can be...			
	Generic products that are naturally gluten-free and can be consumed without concern	964	74.5
	Conventional products where you should check the label	266	20.6
	Specific gluten-free products that can be consumed without concern	64	4.9
Margarines, pork rind, sunflower seeds, flavoured infusions, cold meats and spices are…			
	Generic products that are naturally gluten-free and can be consumed without concern	20	1.5
	Conventional products for which the label must be checked	1235	95.4
	Specific gluten-free products that can be eaten without worrying about it	31	2.4
	No response	8	0.7

## Appendix C

**Table A3:** Results of the questionnaire Q3 to measure the knowledge about the GFD of healthcare professionals distributed by SEEC (*n* = 346).

Question		Number of Responses	Percentage (%)
Which of these pseudocereals do you think may contain gluten?			
	Quinoa	37	10.7
	Amaranth	24	6.9
	None	285	82.4
If a product does not bear the specific “gluten-free” label, but its ingredients do not contain any gluten-containing raw materials and there are no declared traces, can it be considered safe?			
	Yes	83	24.0
	No	211	61.0
	I do not know	52	15.0
Declaring traces of wheat, or cereals containing gluten, is mandatory			
	Yes	245	70.8
	No	46	13.3
	I do not know	55	15.9
Do you recommend the consumption of oats?			
	No, never	166	48.0
	Yes, from the first year of the gluten-free diet onwards	12	3.5
	Yes, from the first year of gluten-free diet and only certified gluten-free oats	101	29.2
	Yes, before the first year of the gluten-free diet, and only certified gluten-free oats	67	19.4
